# A General-Purpose Distributed Analytic Platform Based on Edge Computing and Computational Intelligence Applied on Smart Grids

**DOI:** 10.3390/s23083845

**Published:** 2023-04-09

**Authors:** Juan Ignacio Guerrero, Antonio Martín, Antonio Parejo, Diego Francisco Larios, Francisco Javier Molina, Carlos León

**Affiliations:** 1 Department of Electronic Technology, Escuela Técnica Superior de Ingeniería Informática, Universidad de Sevilla, 41012 Sevilla, Spain; toni@us.es; 2 Department of Electronic Technology, Escuela Politécnica Superior, Universidad de Sevilla, 41011 Sevilla, Spain; aparejo@us.es (A.P.); dlarios@us.es (D.F.L.); fjmolina@us.es (F.J.M.); cleon@us.es (C.L.)

**Keywords:** edge computing, smart grid, data integration, genetic algorithm, particle swarm optimization

## Abstract

Currently, in many data landscapes, the information is distributed across various sources and presented in diverse formats. This fragmentation can pose a significant challenge to the efficient application of analytical methods. In this sense, distributed data mining is mainly based on clustering or classification techniques, which are easier to implement in distributed environments. However, the solution to some problems is based on the usage of mathematical equations or stochastic models, which are more difficult to implement in distributed environments. Usually, these types of problems need to centralize the required information, and then a modelling technique is applied. In some environments, this centralization may cause an overloading of the communication channels due to massive data transmission and may also cause privacy issues when sending sensitive data. To mitigate this problem, this paper describes a general-purpose distributed analytic platform based on edge computing for distributed networks. Through the distributed analytical engine (DAE), the calculation process of the expressions (that requires data from diverse sources) is decomposed and distributed between the existing nodes, and this allows sending partial results without exchanging the original information. In this way, the master node ultimately obtains the result of the expressions. The proposed solution is examined using three different computational intelligence algorithms, i.e., genetic algorithm, genetic algorithm with evolution control, and particle swarm optimization, to decompose the expression to be calculated and to distribute the calculation tasks between the existing nodes. This engine has been successfully applied in a case study focused on the calculation of key performance indicators of a smart grid, achieving a reduction in the number of communication messages by more than 91% compared to the traditional approach.

## 1. Introduction

Nowadays, different infrastructures are based on distributed environments with edge computing, including smart cities [1,2,3,4], smart grids [5], smart buildings [6,7], etc., on a macro scale, or electric vehicles [8,9] and battery management systems on a micro scale. The commercial solution for batteries in current electric vehicles (EVs) is based on different nodes with edge computing capabilities. Usually, these infrastructures need the integration of information in a centralized architecture (big data infrastructure or a machine with enough computing resources) to perform distributed analytic algorithms, which are usually based on regression, clusterization, or classification. Even in big data infrastructures, it is very difficult to apply modelling techniques, which involve complex equations, with dependencies between different data sets. In these cases, a deep study of equations is necessary to provide an algorithm that could apply the equation to a data set or distributed data set. Additionally, the critical nature of information, the communication channel, and the computing capacity of the edge computing nodes establish some constraints that must be considered in the research or study of the equation. This type of problem is usually solved by creating a middleware layer to integrate information and communication in the distributed environment, such as FIWARE, which provides an open data infrastructure with big data analytic capabilities. Thus, in addition to the distributed infrastructure, it is necessary to have a big data or middleware platform to perform the storage and analytics, reducing the responsibility of nodes to sensing and some edge computing tasks. This is one of the most important factors that enables the transition from traditional power grids to smart grids [10].

In the case of the critical nature of information, such as critical infrastructures, it is very difficult to share or transmit the information to other nodes, and it is necessary to have a hard anonymization process or to increase edge computing capabilities, which can increase the cost of edge nodes. Additionally, in these cases, when anonymization is applied, there is a massive use of the communication channel, and it requires that the process run in the same way to all information involved in the process, even if it is not critical information.

Thus, the two main approaches that are usually applied to address the described problems (which are the motivation of the proposed platform) are the following:

- Centralized (or traditional) approaches. In this case, the information is integrated into a common repository [11]. There are several common problems associated with most of the current platforms:
  ○ Information must be integrated into a common repository to perform analytics, usually based on big data. In the case of big data infrastructures, the main algorithms are based on clustering, classification, and regression.
  ○ Integrating information into a common repository involves several privacy and confidentiality problems, which is a very important issue in critical infrastructures. For example, ref. [12] provides a comparison of the privacy preservation problem between a centralized approach and some distributed approaches.
  ○ The implementation of extraction, transformation, and load (ETL) applications is one of the most expensive processes in the modelling process. Ref. [13] is an example of this case, and this integration platform is based on the combination of Flume and Kafka, configuring specific ETLs to integrate consumer information.
  ○ Communications in this case are overloaded in the acquisition and integration of information stages. Sometimes, it is a problem related to the communication infrastructure that could not support massive data transmission; in this case, it is necessary to establish complex infrastructures to centralize the information [14].
  ○ Usually, the anonymization process is developed for each solution, to grant privacy preserving and security access.
- Distributed approaches. In this case, the information is distributed in different nodes in a connected network and is commonly used in industry 4.0 with the Internet of Things (IoT) [15]. Although they provide some advantages in comparison to centralized approaches, current platforms frequently present some common problems:
  ○ The current solutions based on distributed approaches are based on generic approaches of modelling algorithms; most of these algorithms are based on clusterization, classification, or regression. Ref. [16] shows an example of this case, implementing multiple linear regression in a distributed environment.
  ○ Some of the current solutions show an application of an algorithm developed for a specific case with specific distributed data resources, for example, ref. [17], which proposed a specific solution based on a sample average approximation (SAA) algorithm.
  ○ Modelling in distributed approaches is usually based on extraction of the required information from a modelling node, increasing the usage of the communication channel. For example, ref. [18] proposed a frequency analysis based on cosine similarity and deep learning.
  ○ The exchanged information must be preprocessed to determine the privacy or confidentiality of the exchanged information.
  ○ The anonymization process should be designed for each case.
  ○ In this case, there exist some cases based on the development of middleware layers, which have the same problems as distributed approaches; for example, ref. [19] establishes an intermediate node to perform the conversion of the IEC61850 protocol.
  ○ There are other approaches based on serverless technology [20], but in this case, a cluster of worker nodes are necessary to support the infrastructure.

Additionally, in many scenarios, some information could not be centralized due to privacy or confidentiality. Examples include information in the health sector, where health records of specific information in pharmacological studies are sensitive, and in the energy sector, where different services and companies cannot provide direct access to sensitive information, such as customer consumption or EV charging patterns, as they can be hacked by a third party [21]. 

Distributed analytics is viewed as one of the fastest-growing areas of research and the development of new applications in distributed data mining. A typical application on this topic is [22], which provides an exact response time analysis for engine control applications consisting of periodic and engine-triggered tasks scheduled in fixed priority. In [23], the authors developed a medium-sized big data engine that improves the performance in map-reduce computing by splitting the analytic into different segments, allowing the engine to process them in parallel using a hierarchical model. Ref. [24] presents a delay-free, auxiliary ordinary differential equation system, with algebraically coupled split boundary conditions. This system characterizes the solutions of the delay differential equation and is used for the synthesis of solutions. In [24], the authors investigate the optimal sensor quantized rules for the distributed decision-making, under a given fusion rule for conditionally dependent and high-dimensional sensor observations. For this purpose, the authors provide a Monte Carlo importance sampling method to reduce the computational complexity of distributed decision fusion. In [25], a mathematical framework is provided for the considered Free Space Optical (FSO) system over a correlated non-identically distributed Gamma-Gamma channel; analytical BER results are derived with and without the preamplifier for a comparative study. Ref. [26] proposes a machine learning approach, based on analytical inference in Gaussian process regression (GP), to locate users from their received signal strength indicator (RSSI) data from uplink in a distributed massive multiple input-multiple output setup. An algorithm for distributing Boolean equations over networks is proposed in [27], while [28] proposes algorithms for solving linear equations in a distributed manner in networks.

In computational intelligence algorithms, [29] proposes a hybrid Genetic Algorithm (GA) for the optimal shape design of an axially symmetric dual-reflector antenna by combining the GA with a Moving Least Square (MLS) algorithm, which enhances the convergence rate and global search performance. In [30], the authors use the adaptive genetic algorithm technique for exploring low-energy crystal structure configurations, with the aim to find new low-energy non-cubic phases with high saturation magnetization. In [31], a detailed methodology is described to obtain a more efficient version of the GA, and a more detailed and accurate description of the flexibility of the flexible operation of the power plants is described. In [32], the authors study whether the Estimation of Distribution Algorithms (EDAs) are robust against a scaling of the noise intensity and present the concept of graceful scaling in which the run time of an algorithm scales polynomial with noise intensity. This article [33] presents an effective optimization procedure to calibrate the deviations of the beam patterns radiated from the phased arrays of antennas, and it proposes a genetic algorithm that allows one to optimize radiation patterns using the actual phase and amplitude states of the radio-frequency components. In [34], a novel genetic algorithm is proposed that employs species differentiation (SD). In [35], a method for designing an optimized cylindrical magnet is presented using the genetic algorithm to achieve homogeneity (for standard magnetic resonance imaging applications).

Many researchers have suggested new techniques for the generic algorithm with Evolution Control and Particle Swarm Optimization (PSO) algorithm. In [36], a clear method has been proposed to obtain the best predictive control weighting of the model. This method searches for the best tune of the MPC cost function weights, while reducing the user burden of weight tuning and receiving user satisfaction feedback to improve the process. In [37], the closed-loop performance of the Voltage-Double Power Factor Correction (VD PFC) converter is examined, using the dynamic evolution control (DEC) scheme. This study [38] investigates the application of a multi-objective genetic algorithm to obtain a set of weighting factors suitable for use in the Model Predictive Torque Control (MPTC) of an induction motor variable speed drive. In [39], the authors propose the use of a neuro-fuzzy system to dynamically determine the strength with which these operators will affect the process of finding the optimal solution. Ref. [40] proposes an improved Quantum-behaved Particle Swarm Optimization (QTPSO) algorithm based on the two-body problem model to solve the problem faced by other quantum-behaved particle swarm optimization algorithms, where they easily converge on local optimal solutions when solving complex non-linear problems. Ref. [41] presents a novel overall distribution Maximum Power Point Tracking (MPPT) algorithm that can rapidly search the area near global maximum power points. This algorithm is further integrated with the Particle Swarm Optimization (PSO) MPPT algorithm to improve the MPPT accuracy. In [42], a Comprehensive Learning Particle Swarm Optimizer (CLPSO) embedded with a Local Search (LS) is proposed to pursue higher optimization performance by taking advantage of CLPSO’s strong global search capability and LS’s fast convergence ability.

Although some progress had already been made in the use of genetic algorithm, to the best of our knowledge, none of the previous work had investigated its application in a Distributed Analytical Engine (DAE). In this sense, this paper presents a new architecture that uses DAE to increase the edge computing in a distributed environment, without the original information being exchanged between nodes. In real environment in which a smart city platform is deployed, companies agree to share information with problems related to anonymization and coherence. The proposed engine provides an alternative to share and transmit information, where only the result of a calculus or equation is shared. This engine decomposes the modelling equation, with a decentralized approach, which is able to implement a modelling engine in a distributed environment. 

The proposed solution can help solve the previously mentioned issues:

- Prevent massive information exchange, select data from different resources, and analyse the metadata from each edge node based on heterogeneous data source integration.
- Prevent the development of middleware layers, including daemons that deal with local and global processing.
- To prevent the development of ETLs, heterogeneous integration of data sources is used to acquire and identify all metadata from each edge node.
- Providing an interface in DAE to apply not only classification, clustering, or regression but also complex equations by using the previously identified information in different resources.
- Providing an additional security level with privacy preserving and confidentiality control based on Edge Computing Daemon (ECD), which automatically controls the anonymization level and reduce the exchanged information.
- Providing a platform for different problems, using the combination of different subsystems, which provides a general-purpose platform.
- Providing a scalable modelling platform due to the possibility to embed the system in a hierarchical or tree structure.

The decomposition is tested with three different computational intelligence algorithms: genetic algorithm, genetic algorithm with evolution control, and particle swarm optimization, and these are compared to the traditional (i.e., centralized) approach of anonymization and calculation on master node. This engine has been successfully applied in the smart grid context for the calculation of key performance indicators, as will be seen in the presented results.

The rest of the paper is organized as follows. In Section 2, an overview of the proposed solution and the system architecture is given. Section 3 analyses the DAE with different subsystem development in DAE. Section 4 presents the development of ECD. Section 5 presents the concept and formulation of the system design proposal and summarizes the performance of the platform and the results of the research. The contributions to this work and areas for future work are presented in Section 6.

## 2. System Architecture

**Figure 1:**
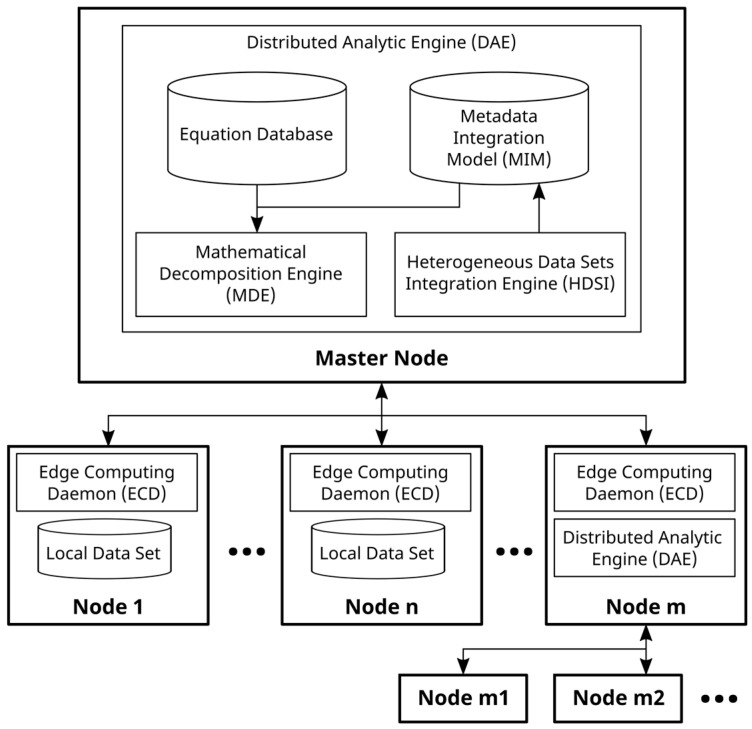
Overview of the general system architecture.

The proposed solution is composed of an engine, installed on a master node, and several ECDs, installed in different data or edge nodes, as shown in Figure 1. The Mathematical Decomposition Engine (MDE) and the Heterogeneous Data Source Integration Engine (HDSI) are the main modules of DAE, which are deployed in the master node. In the edge nodes, the ECD is installed. This daemon is configured to access data for local databases or data sets in each edge node, and it includes the information of master node address. The DAE sends commands to the different nodes to calculate the value of the proposed equation or mathematical expression. 

The case of Node m shown in Figure 1 is an important case in which the node acts at the same time as the worker and master node. Thus, this node could apply the algorithms again to determine another level of decomposition. This approach allows for a highly hierarchical scalable solution. 

Integration of DAE with other applications is achieved by using a Web Service (WS) interface based on REST and JSON. ECD and DAE have this interface available, simplifying integration activities and compatibility problems. Because DAE and ECD have several modules, the DAE platform was developed initially on python and some other modules in Java (in case of HDSI), and the edge computing nodes were developed in Java; however, currently, all the infrastructures are programmed in Python. The system is developed by modules, so each module could work independently.

An efficient and accurate execution allows for distributed analytics, involving all the information that the specific equation needs. This decomposition or division of calculation steps is stored in the equation database, eliminating the need to repeat this decomposition process. Furthermore, if this equation in subsequent analysis is part of a larger equation, this part of the equation is already divided into efficient and accurate steps, thus minimizing computation time and taking advantage of edge computing. 

## 3. Distributed Analytic Engine (DAE)

When a new edge node is added to the infrastructure, the ECD sends a notification to the master node. After this notification, the HDSI engine checks the availability of the node and the information registered about the node. If the node has not been previously registered, the HDSI engine sends a request to collect metadata from this new edge node. Metadata information pertains to the structure of tables or data sets (referred to as technical metadata), and data include the type of value and the statistical analysis of value variation for different components of the data set. Thus, this system provides a privacy preserver in the case of sensitive information, such as in the case of customer consumption information, because all columns of data sets are analyzed separately. In fact, the main objective of this platform is to provide a method of sharing sensitive information with a high level of privacy preservation. Thus, the HDSI engine adds the metadata information to the Metadata Integration Model (MIM). The size of the metadata depends on the size of the data source located in the edge node. The complete process and the metadata involved are specified in [43]. The MIM provides information for the MDE, adding information from the edge nodes, but at metadata level.

### 3.1. Metadata Integration Model (MIM)

**Figure 2:**
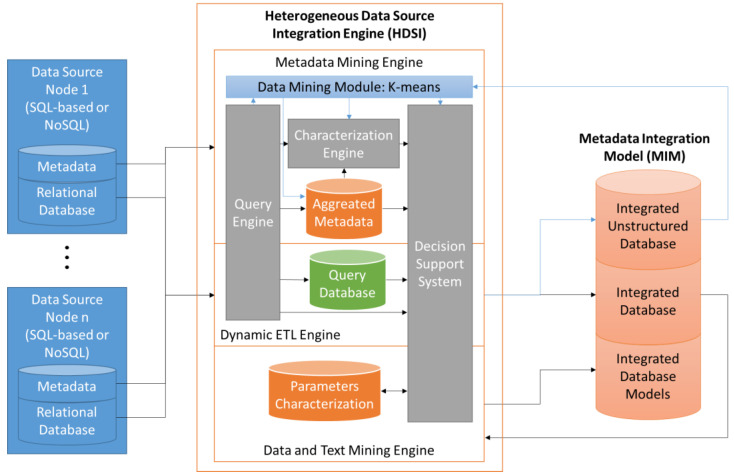
Overview of the internal architecture of HDSI and its connections with the data sources and the MIM.

The MIM integrates metadata from different edge nodes and is extended from the previous work described in [43]. The HDSI version included in this framework includes the integration of unstructured data, updating the platform with some other techniques based on K-means and text mining. The structure of this module is shown in Figure 2. Thus, the DAE has a map of information contained in each edge node, including information about the content of each column or data set. Each module works with the same information in different ways, extracting different characteristics from the information. Metadata mining engine treats metadata from structured and unstructured data sources, the unstructured data sources are treated by the data mining module based on the K-means algorithm. This module classifies the information according to the data from the characterization engine. The dynamic ETL engine implements the extraction, transformation, and load of information, based on characterization and data source. Data and text mining engine is used for the analysis of data content in each column, extracting statistical information and identifying the foreign keys. The result of the integration of heterogeneous data sources is stored in MIM, including structured and unstructured metadata, and the user could configure if the integration of data is performed physically or only using the metadata. The HDSI is described in [43], providing details about the complete process and the role of each module.

For example, the HDSI gathers metadata from each edge node, sending different queries; these queries provide information about the column (structured database) or data set (unstructured database). The meta-information could be divided into two sections, the metadata provided by the system tables of the LDMS and the metadata provided by queries to check the values for each column or data set. Thus, the meta-information from the system tables of the LDMS includes general aspects:

- Node name and address.
- Data structure: structured or unstructured.
- Database name: name of the database in the LDMS.
- Name of different tables and data sets within the specified database name.

For each column of each table (structured data) or element of each data set (unstructured date):

- Data type and length: for example, integer, real, double, etc. It depends on the local database. If there are no lengths assigned to the data in the system tables, this is usually the default length. In the case of complex types, such as double, this length includes the decimal length.
- In case of enumerated data, the different values of enumeration.
- Default values.
- Some other properties that are described by a Boolean value: primary key, foreign key, unique, nullable, empty values, etc. In case of foreign key, the column or element name is stored in the other data.

Additionally, using specific queries designed to grant anonymization of information, some other information is gathered from the available values from the column (structured data) or element (unstructured data):

- Real data length: real data length registered in the data, checking the information stored in the LDMS.
- The different values of the column or data set.
- The frequency of each value.
- For each column or data set, the absolute and relative frequencies of each value.
- Several statistical information about the distribution of values in the column or data set, for example, granularity or regularity, number of errors or nulls, etc.
- Mask: This part of the system is based on fuzzy algorithms. The algorithm infers the format of a data column or set, creating a mask in which all possible values can be fitted.

In the case of structured data, the query is in SQL and is the following: 

SELECT COLUMN, COUNT(*) FROM TABLE GROUP BY COLUMN;

This query returns a table with two columns: the first column is the possible values of the column, and the second column is the number of appearances. This query is run for each column of each table only for the first time. The result of the analysis is performed on the edge node, returning only the information previously described.

This operation is transparent for the final user, and it is automatically run in the platform. The ECD notifies to the MDE any new change in the metadata in the LDMS. Additionally, if the edge node has enough computing capacity, the characterization engine is included in the edge node, to reduce the communication and computation requirements.

### 3.2. Mathematical Decomposition Engine (MDE)

**Figure 3:**
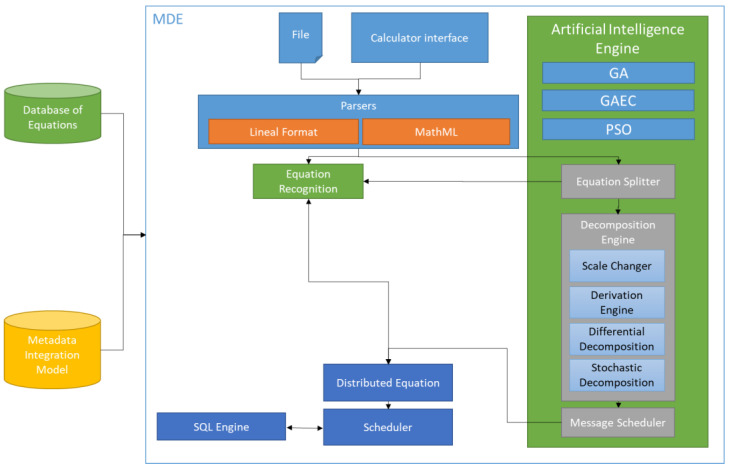
Master node architecture, Mathematical Decomposition Engine (MDE).

The MDE does not have access to the original information; instead, it only utilizes the available parameters and the type of data associated with them. Therefore, the MDE could specify equations that involve any parameter from any edge node, but it only has access to the result of the equation. The equation is introduced into MDE via a web interface (Figure 3), in which the equation is specified in traditional linear format (similar to a calculator interface), MathML (only for files), or OpenMath (only for files). The equation input interface allows the client to protect the equation and to avoid the modifications. When someone attempts to modify a protected equation, a new equation is generated. Additionally, the user can introduce any equation using the buttons (similar to a calculator interface) or directly writing the equation with the specified symbols. The user could include parameters from the MIM. These parameters are shown in groups below the interface. All these parameters have been previously collected from all edge nodes to establish a common base of information. The MDE has a parser for the interpretation of equations in lineal and MathML formats.

The Mathematical Decomposition Engine (MDE) shown in Figure 3 is located in the master node and has several modules. Each of these modules takes advantage not only the mathematical tools but also the meta-information about different columns and parameters, which include the possible values. This module follows the process depicted by the arrows shown in Figure 3:

1. The equation is provided in a standard or plain text format that includes the columns and parameters of HDSI.
2. The parsers apply a grammar to validate syntactical and semantics of the equation, adding metadata to different parts of the equation with information about the type of equation and the dependencies according to the data source locations.
3. The equation is analyzed by the equation recognition module. This module checks the database of equations to find the original or similar equation. This database stores information on all previously decomposed equations or equations that could implement similar models. If the equation is not recognized, it is sent to the equation splitter module.
4. The equation splitter module decomposes the equation into different parts according to the mathematical equation and the metadata previously added, considering the priority of mathematical and logical operators: arithmetic operators (functions, square brackets, brackets, ^, *, /, integer division, module, +, and −) and logic operators (brackets, not and, or, <,>,>=,<=,!=, and ==), and identifying the parts of the equation that could be calculated separately.
5. Thus, if the equation is split into two or more parts, the operation to join the different parts is stored as part of the equation metadata. Thus, each of these parts of the equation is sent separately again to the equation recognition. Parts that were not recognized are sent to the decomposition engine. In case of data with a low variance range, the Pearson correlation coefficient, shown in Equation (1), is based on possible values ($r_{x},r_{y})$ and frequency of values (n) stored as part of the metadata.
  (1) $$r_{p}=\frac{n\sum r_{x}r_{y}-\sum r_{x}\sum r_{y}}{\sqrt{\left[n\sum r_{x}^{2}-{\left(\sum r_{x}\right)}^{2}\right]}\left[n\sum r_{y}^{2}-{\left(\sum r_{y}\right)}^{2}\right]}$$
  In case of an element with a wide variation range, the Spearman correlation coefficient, shown in Equation (2), is used to determine the applicability of the scale changer module, obtaining the same possible value intervals as $r_{p}$. From the point of view of interpretation, both coefficients are the same, but the Spearman correlation coefficient is usually applied when the data have a small quantity of external values.
  (2) $$r_{s}=1-\frac{6\sum {\left(r_{x}-r_{y}\right)}^{2}}{n(n^{2}-1)}$$
  In both cases, the values of the ranges are placed according to the numerical order of the corresponding column. Thus, this type of decomposition is usually applied to structured data. The value of these correlation coefficients, the equation structure, the number of edge nodes involved in the equation for each split, and equation metadata apply different modules with different functionalities:
  - Scale changer is a module that applies different changes on the equation that are equivalent to the original equation. Different techniques are applied, depending on the type of equation. This module has a rule database equivalence with 211 rules, which attempts to split the equation into equivalent one, usually focused on the parameters without correlation. In case of an arithmetic equation, logarithms (in case of equation based on power variables), trigonometric equivalences, change of variables, aggregation, finite differences, etc., are applied. In the case of the logic equation, Boolean algebra is applied. The granularity or regularity of the measurements could provoke the application of this module to adapt the granularity between different parameters, based on the metadata mining performed by the HDSI module.
  - The derivation engine is a module based on decomposition that treats different parts of the same equation. In this case, the equation represents a curve that will be decomposed into different parts, applying integration and derivation to determine the different sections of the curve. Integration is based on adaptive quadrature [44]. The derivation is applied using a numerical analysis technique, based on the forward difference (3), backward difference (4), and the central (5) difference, supposing $f\left(x\right)$ as the split equation. In these equations, the x parameter is replaced by the parameter that the system cannot extract from the node due to security constraints or the difficulty to perform a correct anonymization. This engine is used when the information cannot be extracted from the edge node, and the edge node has no privileges to run the result; in this case, the result is an approximation due to the constant term of derivation.
    (3) $$\frac{df(x)}{dx}\approx \frac{f\left(x+h\right)-f(x)}{h}$$
    (4) $$\frac{df\left(x\right)}{dx}\approx \frac{f\left(x\right)-f(x-h)}{h}$$
    (5) $$\frac{df\left(x\right)}{dx}\approx \frac{f\left(x+h\right)-f\left(x-h\right)}{2h}$$
  - Differential Decomposition is a module based on the application of numerical methods to differential equations based on the Runge–Kutta method [45]. In this case, the correlation coefficients should be in the interval [−1, −0.9] or [0.9, 1]. The Runge–Kutta method is based on Equation (6) and Equations (7) and (8), which define the numerical method. In Equation (6), Ω is an open set with the condition of the initial value of f, where $\left(t_{0},y_{0}\right)\in \Omega$. Equation (7) is the generic equation, where *s* is the order, $∆t_{n}$ is the step in each iteration of the Runge–Kutta method, this means the increment between *t_n_* and *t*_*n*+1_. Equation (8) represents terms of intermediate approximation, where $a_{i,j}$, $b_{i},and\;c_{i}$ coefficients are based on adaptive quadrature [44]. However, these equations are general methods, but Runge–Kutta is a set of iterative numerical methods [45]. This module is applied only to parameters with a high correlation rate and is usually applied in a single split of equations with parameters from different edge nodes.
    (6) $$y^{\prime }\left(t\right)=f\left(t,y\left(t\right)\right)\;with\;f:\Omega \subset R\times R^{n}\to R^{n}$$
    (7) $$y_{n+1}=y_{n}+∆t_{n}\sum_{i=1}^{s}b_{i}k_{i}$$
    (8) $$k_{i}=f\left(t_{n}+∆t_{n}c_{i},y_{n}+∆t_{n}\sum_{j=1}^{s}a_{i,j}k_{j}\right)\;i=1,\ldots ,s$$
  - Stochastic decomposition is a module that employs stochastic numerical methods to estimate different equivalences to the equation or part of the equation [46].
6. The Message scheduler designs the message exchange pattern to execute the different parts of the equation in the ECDs. Based on the information involved in each part of the equation, the Scheduler designs the request message flow, determining whether it is necessary to exchange anonymized information (only anonymized information is exchanged) or processed at the edge node.
  The Message scheduler module is a special case in the decomposition process. There are special cases where the equation cannot be decomposed by the proposed system and needs to make the calculation step in the master node. In this case, the DAE fixes the identifier field and the parameters involved, sending the information to each ECD. Each ECD applies a SHA-2 of 512 bits to each identifier field value, associating the requested parameter. Thus, the information is sent along with an associated hash value. Each node that needs to cross the information from different resources will check the hash. Therefore, the system does not need to maintain a general index correspondence table with all identifier records. In some cases, in which the identifier structure is more complex, a distributed Merkle hash tree [47] can be created using the same SHA algorithm. The distributed Merkle hash tree is not treated in this paper.
  This approach increases the information exchanged between the master and edge nodes, decreases the load of the edge computing nodes, and increases the load of the master node and the communication network.
7. The distributed equation module designs and implements the process of aggregating all information from each ECD, following the instructions stored in the equation metadata.
8. The Scheduler with the support of Statement Query Language (SQL) Engine manages the process, sending instructions to each ECD involved in the process, and retrieving the results, which are sent back to the Scheduler.

Modules 5.b and 5.c were not used in any of the solutions obtained for the proposed case study, which will be presented in Section 5, although some of the generated and invalid individuals (i.e., potential solutions for the decomposition of equations that were generated but finally were not selected) included these modules.

### 3.3. Artificial Intelligence Engine (AIE)

The AIE is based on two artificial intelligence approaches that are tested: genetic algorithm and swarm intelligence. In the case of genetic algorithm, the algorithm used is a genetic algorithm GA and GAEC. GAEC provides an additional control over the generation of new populations. In case of swarm intelligence, the algorithm used is a particle swarm optimization (PSO) algorithm. PSO is used to simplify the mathematical expression according to the data. The decomposition engine is executed depending on the capabilities of the platform. 

In all algorithms, the fitness evaluation is based on the number of messages and the computational cost of the edge nodes. 

#### 3.3.1. Genetic Algorithm (GA)

**Figure 4:**
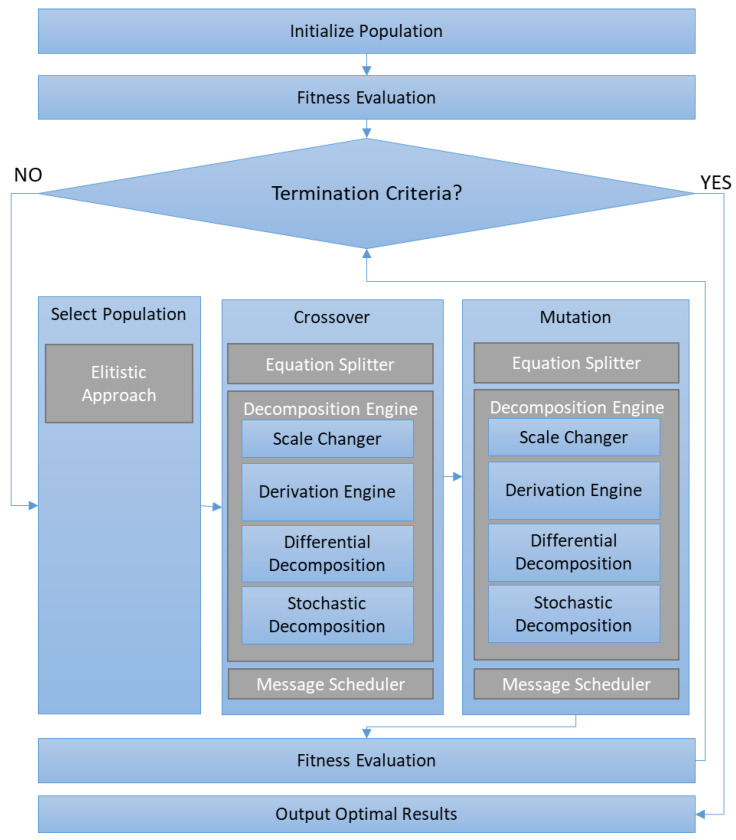
Diagram flow of the Genetic Algorithm.

GA [48] is a bioinspired algorithm based on the search for a solution in a population, where everyone represents a possible solution to the proposed problem. Each individual is evaluated according to proximity to the solution using a fitness value. Each iteration of a new population is generated based on an elitist strategy; this means that a selection operator takes the best individuals (with the best fitness values). These individuals are mixed with other individuals by using the crossover operator, or some of them are modified with mutation operator. These operators have associated a probability following the values suggested in [49]. In this case, the operators were selected to create a high level of independence from the population, whose individuals depend on the complexity of the equation. Additionally, another level of probability is added to the crossover and mutation operators to select between each possible modification of the equation. After the application of these operators, a new population is generated and evaluated again. If the stopping criteria (the best solution is obtained with the fitness threshold) are reached, the solution is found in one or more individuals of the population. If the stopping criteria are not reached, the process starts again. A flow diagram is included in Figure 4. 

#### 3.3.2. Genetic Algorithm with Evolution Control (GAEC)

**Figure 5:**
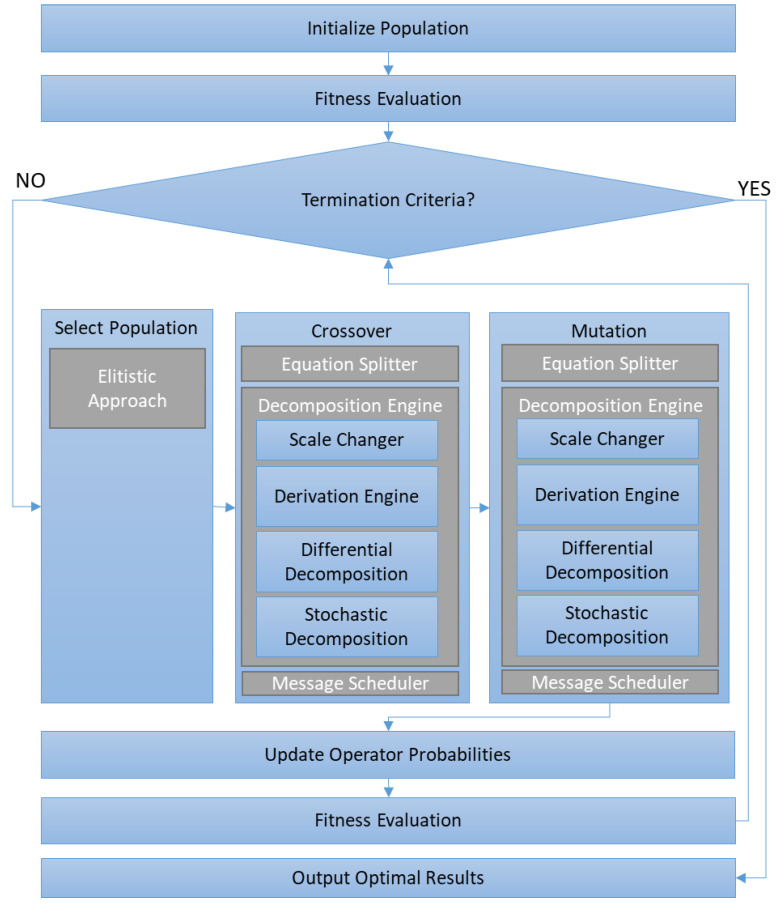
Diagram flow of Genetic Algorithm with Evolution Control.

Evolution is controlled by the fitness curve. In each population, individuals are evaluated. The best fitness is collected to build the fitness curve. The GAEC takes advantage of this curve, taking the slope of the previously indicated point in the fitness curve. This slope stabilises the probability of crossover and mutation operators. The crossover operator is associated with the sine of the slope angle. The mutation operator is associated with the cosine of the slope angle. In this case, similar to GA, operators based on the slope angle of the fitness curve provide independence from the population. If the stopping criteria are reached, the solution is found in one or more individuals in the population. If the stopping criteria are not reached, the process starts again. Figure 5 shows a diagram flow. The authors did not find any reference that proposes the application of this algorithm with dynamically modified probability based on trigonometric functions. Although there are authors that provide similar optimizations in the genetic algorithm, [50] provides an approach to optimize the algorithm with a sine cosine algorithm and [51] provides several functions for adaptive probability calculation for a global function probability for operators, but the function is not exactly the same and adds some optimization to the probability calculus. Thus, the proposed approach is a simplified application and reduces the computational cost.

#### 3.3.3. Particle Swarm Optimization Algorithm (PSO)

The algorithm employs a PSO [52] to stabilise the new decomposed equation. The canonical PSO model consists of a swarm of particles, which are initialized with a population of random candidate solutions. They iteratively move through the problem space of dimension *d* to find new solutions, where the fitness can be calculated as the measure of certain qualities. Each particle has a position represented by the position vector *x_id_* (*i* is the index of the particle, and d is the dimension) and a velocity represented by the velocity vector *v_id_*. Each particle remembers its best position in the vector *x_i#_*, and its *j-th* dimensional value is x_#ij_. The best position vector among the swarm is stored in the vector x_*_, and its *j-th* dimensional value x_*j_. At iteration time *t*, the update of the velocity from the previous velocity to the new velocity is determined by Equation (9). Then the end position is determined by the sum of the previous position, and the new velocity is determined by Equation (10).

*v_id_*(*t* + 1) = *w · v_id_*(*t*) + *c*_1_
*· ψ*_1_
*· (p_id_(t) –x_i_(t)) + c*_2_
*· ψ*_2_
*·* (*p_g_*(*t*) − *x_id_*(*t*)), (9)

*x_id_* (*t* + 1) = *x_id_*(*t*) + *v_id_* (*t* + 1) (10)

where *c*_1_ and *c*_2_ are constant weight factors, *p_i_* is the best position achieved by particle *i*, *p_g_* is the best position obtained by the neighbours of particle *i*, *ψ*_1_ and *ψ*_2_ are random factors in the interval [0, 1] and w is the weight of inertia. Some references denote *c*_1_ and *c*_2_ as the self-recognition component and the social component coefficient, respectively. After several tests, the self-recognition component is set to 1.2, and the social component coefficient is set to 1.7.

#### 3.3.4. Convergence of Algorithms

The study of convergence and avoiding stagnation at local optima is from another research line, and it will be out of scope of this paper. However, a discussion on this topic is included below. 

In the first place, the proposed algorithms are designed to run on DAE, so these algorithms only work on master nodes. Thus, the major processing is focused on the master nodes and is executed in two situations: when the final decomposition is stored in the equation database or when adding/removing resources from the network that involve the data in the equation.

In the case of proposed algorithms, a balanced application of diversification and intensification strategies is applied to avoid the potential stagnation in local optima. To allow this balanced application, all algorithms implement an additional mechanism that stores all discarded solutions, and with each solution, a hash is generated and stored. This mechanism allows one to implement a diversification strategy, removing solutions that could cause stagnation of the algorithm. This technique and the constant values mainly avoid stagnation of the PSO algorithm. The self-recognition component controls the weight of the best position obtained by the particle. Therefore, in this case, a high value means that a particle will have an intensification strategy, and a low value means that the particle will have a diversification strategy. A value of 1.2 provides a diversification strategy. Furthermore, the social component adjusts the influence of the neighbouring particles on the current particle. A high value for this coefficient means that a particle has a greater tendency to follow the solutions in neighbouring particles (intensification strategy), while a low value means that the particle does not follow the neighbouring particles (diversification strategy). A value of 1.7 gives an intensification strategy. The test shows that this combination of values optimizes the algorithm, reducing the risk of stagnation in local optima.

Both GA and GAEC provide additional techniques to avoid the stagnation at local optima based on mutation operator and the probabilities assigned to each operator. In the case of GA, the assignation of probability to each operator, the selection operator strategy, and the discarded solutions provide the balance between diversification and intensification strategies. In case of GAEC, an additional technique is included providing a dynamic probability for crossover and mutation operators. Thus, when a set of solutions have been explored, the probability assigned to operators provides an intensification strategy because the slope of fitness curve is higher than in case of local optima. In contrast, when the slope of the fitness curve approaches zero and there are a lot of discarded solutions, a local optimum is detected, so the probability of mutation will be increased, providing a diversification strategy. However, the integration of memory to store information about discarded solutions provides a common point to compare between algorithms. 

## 4. Edge Computing Daemon (ECD)

**Figure 6:**
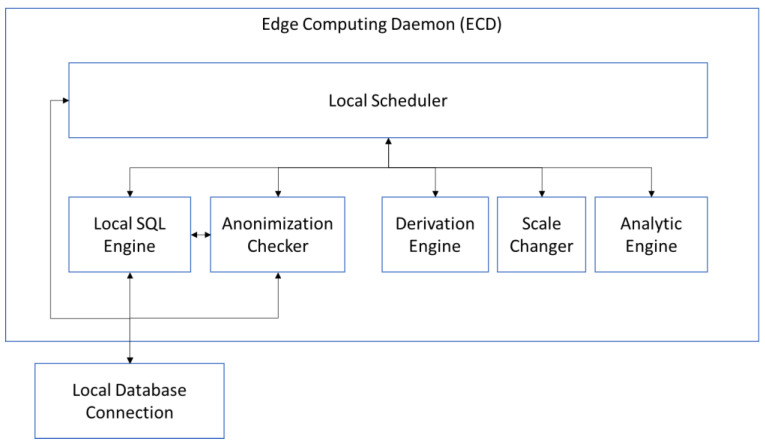
Overview of the ECD architecture.

The local ECD (Figure 6) is synchronised through the local scheduler, which is responsible for information exchange. The local SQL engine is usually provided by the Local Database Management System (LDBMS), but in some cases, an adapter is necessary to understand the content of messages, translating messages to the local SQL engine. The anonymization checker performs five tests to check the anonymization level before the SQL is launched. These tests check several levels of anonymization: direct identifiers, indirect identifiers, pseudonymous or encrypted data, untraceable sensitive personal information, and untraceable sensitive business information. These tests guarantee the operation of anonymized information and work over structured and unstructured data, for example, to check the information included in a text area. In case one or more tests are not successfully applied, several rules are applied to attempt to rectify the SQL query. If the rules are not successfully applied, the SQL query is returned with an error code, and the DAE decomposes the equation again, updating the fitness values and weights. 

When the request includes decomposition tasks, the ECD has available a derivation engine and a scale changer. Both modules oversee mathematical decomposition, and if necessary, they provided the equation required by the MDE. Additionally, an analytic engine is available, which includes model local information, to share the model instead of the original data (thus preserving privacy). The ECD works directly with the LDMS. 

**Figure 7:**
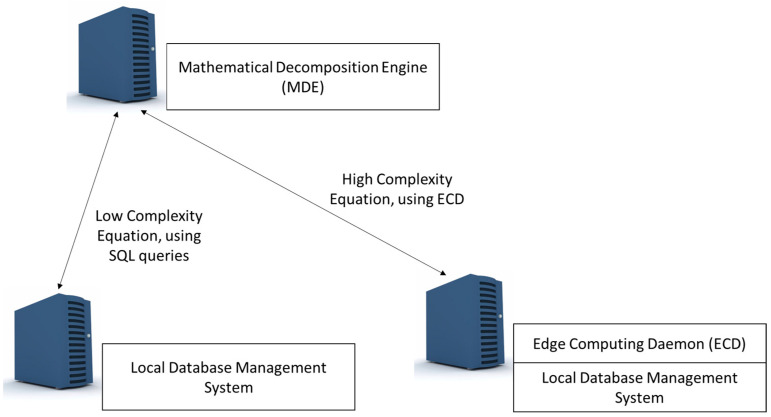
Strategies for distributed data mining.

The ECD could directly access the local database or data source when the complexity of the equation and the information involved are not sensitive and the extraction process is safe. This security check is conducted according to the metadata information. The metadata mining applied by HDSI identifies fields with sensitive or critical information. In case of critical information, in edge nodes with enough resources, the analytical engine provides the result of analysis of metadata (according to the edge node, it could include the characterization engine module), sending only the processed information. Thus, there are two strategies to extract information from the edge nodes (Figure 7). One strategy is used on high-complexity equations, which use the different functionalities in the ECD. The other strategy is only used in the case of low-complexity equations, which could be solved with a SQL query. For example, if the MDE needs the average value of a parameter, the ECD will send the SQL query to the LDMS and send back the response to the MDE.

## 5. Experimental Results

The test is performed to validate the platform in a distributed context, specifically in a smart grid context with smart city KPI monitoring system. In this case, the proposed platform is a system to perform the calculation to create a data warehouse that involves different information resources. Information and architecture are specified in [53]. The KPI monitoring system is used to assess a smart grid project goal. In this case, the architecture is based on a master independent node and four edge nodes. The smart-city KPI monitoring system is deployed in the master node, including DAE. The ECD is deployed in every edge node, representing different systems: consumer portal, meteorological data portal, lighting system, data acquisition system, and vehicle to grid (V2G) management system. Each of these systems stored the information in Common Information Model established by IEC in the 61970 and 61968 international standards. In this case, the equations are modified for the I_1_ to I_21_ indicators described in [53]. The KPI monitoring system has a forecasting module available, which provides an estimation of indicator values according to different scalability parameters. Thus, the indicator equations not only involve information from different columns but also include the equations of the forecasting models, which involve between seven to fourteen different dimensions, according to the number of parameters that can be configured to get a forecasting value. The indicators involve the following KPIs:

- Reduction in Overall Demand
- Reduction in Peak Demand
- Reduction in Technical Losses
- Reduction in Street Lighting Consumption
- Reduction in High-Power Customers’ Consumption
- Reduction in Domestic and Small Business (SME) Consumption
- Influence on Vehicle-to-Grid technology (V2G)
- Improvement in the Efficiency of the Control System
- Percentage of Total Renewable Generation
- Percentage of Renewable mini-Generation
- Percentage of Renewable micro-Generation
- Reduction in CO_2_ emissions
- Improvement in Zonal Quality
- Improvement in Waveform Quality
- Improvement in Early Detection of LV Occurrences.
- Improvement in Response to MV Occurrences
- Extension in the Service Life of Transformers
- Extension in Service Life Switches
- Extension in Service Life Cabling
- Reduction in Breakdown Costs
- Reduce maintenance costs.

The proposed DAE system has been deployed in the described scenario, performing the automatic integration of metadata from the different resources. Once the metadata are collected by DAE and gathered from ECDs, the metadata are analysed, and the parameters are identified. Thus, the DAE is available for the specification of new equations.

To show the functionalities of the platform, the final equation of indicator 1 or the reduction in the overall demand is included below, decomposed into different stages. This indicator shows the reduction in the overall demand of a smart grid, from the substation point of view per day. In the paper, four equations are defined, and the final equation after replacing all equations is shown in Equation (11).

(11) $$I_{1}\left[\%\right]=\frac{f\left(temp,work\right)\frac{\sum_{j=1}^{24}\sum_{m=1}^{6}D_{baseline,h,m_{j}}\left[kW\right]}{24}-f\left(temp,work\right)\frac{\sum_{j=1}^{24}\sum_{m=1}^{6}D_{h,m_{j}}\left[kW\right]}{24}}{f\left(temp,work\right)\frac{\sum_{j=1}^{24}\sum_{m=1}^{6}D_{baseline,h,m_{j}}\left[kW\right]}{24}}$$

In this equation, *j* is the hour of the day, *m* is the power line (in the proposed model there are 6 lines), so *D_h,mj_* is the demand of the line m and at hour *j*, *D_baseline,h,mj_* is the baseline demand studied from previous data of the line m and at hour *j*, and *f(temp, work)* is a constant demand modifier according to weather conditions and the work calendar. In the proposed example, all the systems have the same location, so this parameter will be a common constant for the three terms and will be eliminated from the equation. The first step is to identify the source of the information of each parameter and the node:

- *D_h,mj_*: this information is in the data acquisition module because the information is at the substation level.
- *D_baseline,h,mj_:* this information is in the Smart-City KPI monitoring system because this information is pre-modelled by such a system.

Thus, the final solution of the proposed equation:

- The edge node, Meteorological Data Portal, is discarded from the calculation by the DAE because HDSI identified a constant value for the current available information. Thus, there are no messages to/from this edge node.
- Edge node, Data Acquisition System, will execute the Equation (12). Thus, there is one message that includes the information on the equation from the master node to the edge node and another message from the edge node to the master node with one number *(<results-from-ECD*>). The traditional approach will send all the information about *D_h,mj_* to the master node.
- The master node, the Smart-City KPI Monitoring System, will execute the Equation (13). There is no exchange of information for this equation because it is the master node, just wait for the reception of <*results-from-ECD*>.
  (12) $$<results-from-ECD>=\frac{\sum_{j=1}^{24}\sum_{m=1}^{6}D_{h,m_{j}}\left[kW\right]}{24}$$
  (13) $$I_{1}\left[\%\right]=1-\frac{<results-from-ECD>}{\frac{\sum_{j=1}^{24}\sum_{m=1}^{6}D_{baseline,h,m_{j}}\left[kW\right]}{24}}$$

The proposed application was initially designed with a centralized acquisition system to store the meta-information from different data sources in a centralized data source. Thus, the acquisition system has been replaced by the DAE; additionally, the original application needs to make a request to the query equation in WS in the DAE. Therefore, although there are several changes in the original software, the cost of infrastructure is reduced because the information is spread over different edge nodes and the modelling or calculation process is distributed, employing edge computing nodes by ECD.

Additionally, this case has an additional interface available to provide new equations that allow the proposed system to improve the functionalities of the original applications.

When the different edge nodes add new information, the master node does not need to add this new information because it already has the metadata and changes in the metadata or structure of the data are uncommon in deployed systems. However, the ECDs check the metadata of their corresponding data sets, and if the metadata are modified or updated with new fields, tables, or structures, the ECD notifies the changed and updated metadata to MDE. Thus, the exchange of information is drastically reduced because there is no acquisition stage. However, the modelling or edge computing stage reduces the exchange of information because only the results of each part of the equation are exchanged when the MDE requests it. In addition, the hard disk space requirements are lower than those of a centralized system.

In this paper, three computational intelligences are compared to reach the best mathematical decomposition of an equation on the DAE: GA, GAEC, and PSO. The mathematical decomposition provided by each of these algorithms provides different parts or steps in the resolution of the equation. The main difference between different approaches is the final decomposition scheme and the number of steps that are decomposed by the scheduler module. Thus, the evaluation of different approaches is supported by several parameters:

- Convergence time (*t_c_*) and Average Convergence time (${\overset{-}{t}}_{c}$). They refer to the time invested in achieving the first optimal solution from the initial population. In this case, it is very difficult to establish an optimal solution because the space of possible solution could be infinite. Therefore, a threshold is established by considering that all the steps of the equation are scheduled and the equation could be solved on the platform. The convergence time is measured in the number of iterations. The threshold is established according to the number of iterations without any changes in the population (in case of GA or GAEC) or particles (in case of PSO).
- Result time (*t_r_*) and Average Result time (${\overset{-}{t}}_{r}$). They refer to the time invested to obtain the result for the proposed equation at the master node. The result time is measured in seconds (s).
- Average Edge Processing time (${\overset{-}{t}}_{p}$). It refers to the average of processing periods at each edge node. This period includes the whole edge computing period in each node: the local query period and the time consumed by the ECD module. The time extracted from the ECD and query in database is added for each node, and the average is the average edge processing time. This parameter is measured in seconds (s).
- Percentage of message reduction (Pm). According to the total message exchanged in the traditional approach, a percentage of messages are implemented for each approach. Message reductions show the percentage difference between the traditional approach and the proposed approaches; therefore, this means the percentage reduction of exchanged messages.

Normally, the convergence time, the result time, and the edge processing time depend on the computational capabilities of the MDE (for convergence and result time) and ECD (for result and edge processing time) nodes. The system is virtualized using 22 cores, 128 GB of RAM, and 10 TB of hard disk drive (HDD) space. Virtualization is configured as one main computer with eight cores, 32 GB RAM, and 1 TB HDD. Three edge nodes were configured with four cores, 16 GB RAM, and 1 TB HDD, and two edge nodes were configured with two cores, 16 GB, and 1TB HDD. The transmission time on the network is not evaluated because the system is running in a virtualized environment. The study of different configurations and the latency related to messages on the communication channel are not the objective of this paper, because the improvement of the proposed approach is based on the reduction of the number of messages and size of data section included in messages, without adding additional security constraints to the existing solutions. Generally, in this approach, only the average values are evaluated because in each KPI, there is a different number of equations calculated simultaneously in each edge node. Thus, in smart grid context, 21 equations are computed for each indicator, including reduction in overall demand, reduction in peak demand, reduction in CO_2_ emission, improvement in zonal quality, extension on service life of transformers, etc. In this case, the main problem is the mathematical decomposition of the equation and the efficiency of this process, because of the large amount of information involved in the process. In the proposed case, the reduction in information exchanged is 96.21%. Furthermore, the response messages only contained aggregated information, increasing the efficiency of the calculation and reducing network congestion. 

Table 1 shows the result of comparing GA, GAEC, PSO, and the traditional (centralized) approach based on centralized master node. The traditional approach is supposed to perform only the metadata acquisition stage, and all equations are solved by scheduling the exchange of all anonymized information. Thus, in traditional approach, all information is anonymized and sent to the master node for the execution of proposed equation. As shown in this table, the best approximation is reached by the PSO approach. In the case of traditional approach, ${\overset{-}{t}}_{p}$ provides the time invested by edge nodes to extract information, which is sent to the master node, and stored in a centralized repository. 

Furthermore, in the presented test (although the system is run in a virtualized environment), the size of the messages is optimized, and the network downtime between request and response is reduced. In the proposed smart grid context, only 15% of the messages contain extracted information from the database, and the rest of the messages only contain aggregated information. 

The average convergence time is calculated only during the first request. Once the equation is decomposed, it is stored in the equation database. Thus, when the system requests this equation next time, the time-consuming calculation does not need to include the average convergence time.

**Figure 8:**
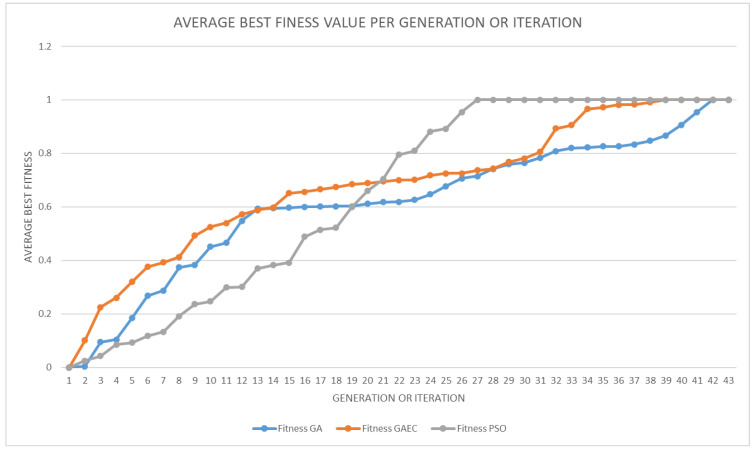
Average best fitness value per generation or iteration.

**Figure 9:**
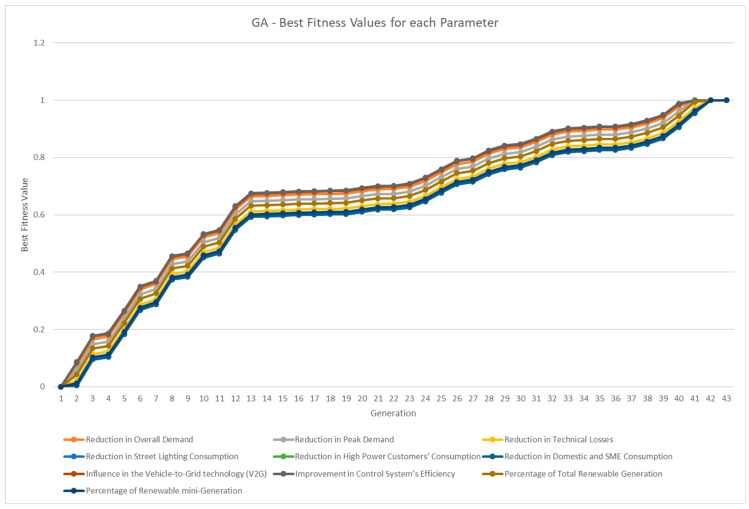
Best fitness values per generation for 10 parameters in GA.

**Figure 10:**
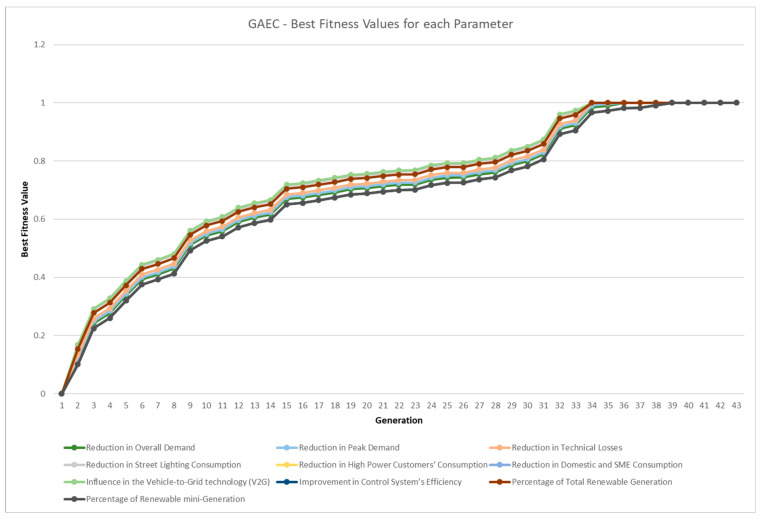
Best fitness values per generation for 10 parameters in GAEC.

**Figure 11:**
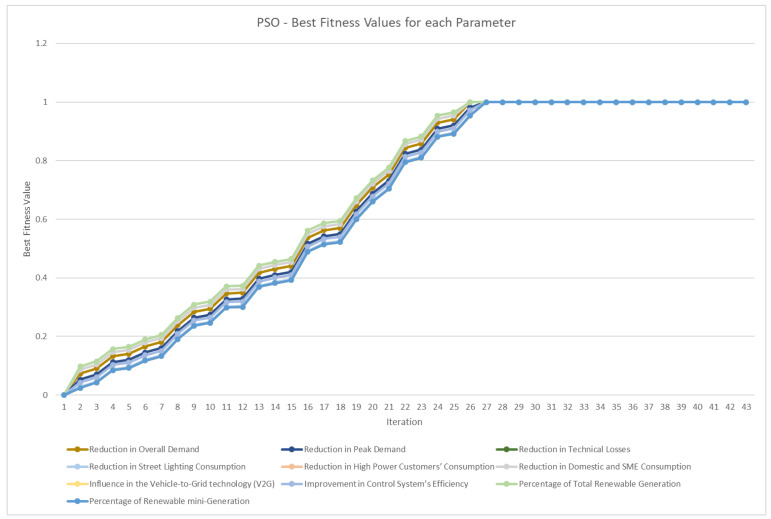
Best fitness values per generation for 10 parameters in PSO.

The fitness function is based on the number of steps divided by the number of binary operators. Thus, when the number of steps is equal to the number of binary operators, this means that the equation is separated at the maximum level of decomposition because it is at the parameter level. The average best fitness value for each generation (for GA and GAEC) or iteration (for PSO) is shown in Figure 8, and it is calculated for each KPI equation. As shown in Table 1, PSO was converged first, but GAEC and GAEC achieved better fitness values in the first generations. This information is more detailed in Figure 9 for the GA approach, Figure 10 for the GAEC, and Figure 11 for the PSO, showing the best fitness value in each iteration for each KPI. One of the future research lines is the inclusion of new techniques to improve the increase in fitness value in each generation or iteration.

## 6. Conclusions

The proposed distributed analytical platform is based on edge computing. The master node has the DAE, which performs the decomposition and scheduling of the equations in one or more steps. Each step is run in the corresponding ECD, returning the results and performing the final calculations. The proposed platform is evaluated in, a smart grid context, in which three CI algorithms, GA, GAEC and PSO, are compared with the traditional approach. All three CI algorithms offered better results than the classical solution. The proposed system decreased the message communication by 96.21% in the case of GAEC. However, in the case of iterations, PSO is the best approximation in terms of convergence, the time invested on the edge nodes, and the time required to achieve the results. Overall, all proposed algorithms provided a communication reduction greater than 91%.

The proposed DAE platform offers several significant contributions to the improvements:

- Reduction and optimization of information exchanged: The optimization of the equation is run once, but it will be optimized again if the distributed data structure is changed, for example, adding new nodes with additional information or duplication. Once the equation is decomposed, the equation and the calculation steps are stored in the database of equations, and it is valid for a determined map of metadata. Thus, this equation is designed to maximize the calculation in the edge nodes, exchanging only the results of the calculus, making the final calculus in the MDE. Therefore, the calculation time is reduced because the calculus is parallelized, and the information exchanged is decreased because the edge nodes send only aggregated information.
- Preservation of privacy using anonymization: Privacy preservation is guaranteed because the original information is never sent.
- Increased analytics capabilities without extending the computational availability of the network: For example, if the network has several edge nodes, a new one can be added without the increase in message traffic due to the functional strategy of the system, which optimizes the equation and reduces the information exchanged.
- Reduced network downtime between request and response: The MDE requests certain calculus and data for ECD. The ECD could optimize the calculus, but usually the calculi are optimized by using SQL query; additionally, the ECD could apply diverse operations provided by different modules integrated in the ECD.
- Simplicity in adding an edge node: It is simple to install the added edge node in the ECD, and the installed edge node provides the credentials to access the local database and the link to the MDE. The system automatically identifies the local database management system and synchronizes with the MDE.
- Distributed analytics through decomposition of mathematical equations from modelling tools: The final user could access the result of a specific mathematical model without accessing the original information, enabling distributed analytics.

## Figures and Tables

**Table 1:** Comparison between GA, GAEC, PSO, and Traditional Approach in the Smart Grid Context.

Algorithm	Pm (%)	${\overset{-}{t}}_{c}$ (Iterations)	${\overset{-}{t}}_{r}$ (s)	${\overset{-}{t}}_{p}$ (s)
GA	91.26	42	0.120	0.123
GAEC	96.21	39	0.070	0.110
PSO	92.31	27	0.020	0.095
Traditional Approach	-	-	1.7	0.021

## Data Availability

Data sharing not applicable.
